# The Field of Science

**Published:** 1893-12-02

**Authors:** 


### The Field of Science.

The Legislative Council of Hong Kong has lately introduced a "Morphine Ordinance" with the object of effectually putting a stop to the practice of morphine administration by unqualified persons. Hitherto Chinese coolies were content to submit to this practice at the hands of any uninitiated operator, many resorts being open to them for this purpose in the large towns.

We learn—on the authority of the *Journal Officiel*—that suicides are increasing in France to a somewhat alarming extent. In 1890 the number of deaths ascribed under this heading in the report, which bears official seal, is put down at no less a figure than 8,410. From the same journal we herewith append a report which, unhappily, speaks only too well for itself:—

| Years. | Average Annual Suicides. | Per 100,000 Inhabitants. |
|---|---|---|
| 1861—65 | 4,661 | 12 |
| 1866—70 | 4,990 | 13 |
| 1871—75 | 5,276 | 15 |
| 1876—80 | 6,259 | 17 |
| 1881—85 | 7,339 | 19 |
| 1886—90 | 8,226 | 21 |

By this record, taken at quinquennial periods, we are forced to admit the steady increase which has existed in the proportionate number of suicides in France during the last thirty years.

Dr. Andrews in his paper, published in the Report of th Medical Department of the Local Government Boaru, states that the virulent anthrax bacillus dies within twenty-four hours when injected into a tube containing pus, and others succumb after a day or two. A few, however, including the diphtheria bacillus, thrive and multiply in this medium. The inquiry, however, opens up a new field of investigation, whose fruits can as yet only be surmised. Work like this often appears at first unpractical, as pure science must, to a certain extent, always be. The discovery of new facts and their application to ordinary circumstances do not always lie in the same hands, and it is the latter only that the public will consciously pay for. Therefore it is the more incumbent that the Government of a country should make possible the first, the essential but unremunerative stage. It can hardly be said that the British nation largely subsidises science, but it does a little, and that little has had most useful results. It is to show some of these results that we have dealt with the medical work of the Local Government Board in a series of articles, the sixth of which appears in the present issue.

The New York Treasury Department has semi-officially declared that all fear of further cases of cholera being introduced through immigrants from Europe being at an end, American surgeons stationed at the principal European ports will now be recalled. This decision was finally arrived at after due consultation with the authorities of the Marine Hospital Service.

Owing to the recent deaths of two children in one family at Elwood, in New South Wales, caused, says the health officer of that place, by using milk from a dirty dairy, there is an outcry at present for the appointment of duly qualified veterinary stock inspection throughout Victoria. This matter was brought up in Parliament in October, and the attention of the Minister of Agriculture drawn to the pressing necessity of action. Hitherto the inspection has been only nomiaal, such appointments having been given to scab-in-sheep inspectors, sometimes ex-policemen, Custom House clerks, or railway men. Last summer, at the annual convention of the Victorian Dairymen's Association, a resolution was unanimously carried demanding that the Minister of Agriculture should appoint competent veterinary inspectors to examine dairies and their surroundings, and offering to contribute, in the shape of an annual licence on the same, the cost of such veterinary inspection.

It has been remarked that when science gets sensational one is apt to scan it a little narrowly. The latest export of "liquid air" has not been received quite credulously in all quarters. But, be that as it may, it is an ascertained fact that a quantity of air liquified through Mr. Dewar's process was not long since despatched from London to Cambridge. This new fluid was conveyed in a double glass flask, so constructed that the inner and outer bottles respectively were fitted with attenuated mercurial vapour and some liquid mercury. By this means the liquid air had a covering of a very fine film of mercury, which was rendered impervious to heat, having a reflecting surface. The flasks were subsequently incased in solid carbonic acid, supplying an envelope 80 deg. below zero. So much for the description of Mr. Dewar's ingenious experiment, and which does not appear to be of any very involved nature. We have yet to be told to what special purpose this can be applied.

